# Association of blood urea nitrogen to serum albumin ratio with mortality in ICU patients with sepsis-induced coagulopathy: A Retrospective Cohort Study from MIMIC-IV database

**DOI:** 10.1097/MD.0000000000048296

**Published:** 2026-04-10

**Authors:** Mei Li, Huibo Wang, Hua Wang, Zhaojun Xu

**Affiliations:** a Department of Critical Care Medicine, Ningbo No. 2 Hospital, Ningbo, Zhejiang, China; b Hebei Medical University, Shijiazhuang, Hebei, China; c Handan Central Hospital, Handan, Hebei, China.

**Keywords:** blood urea nitrogen to serum albumin ratio, critical care, mortality, sepsis-induced coagulopathy

## Abstract

The blood urea nitrogen to serum albumin ratio (BAR) has shown promise as a prognostic marker in sepsis, yet its specific role in sepsis-induced coagulopathy (SIC) remains unclear. We aimed to clarify whether BAR informs mortality risk in critically ill intensive care unit (ICU) patients with SIC. Using the Medical Information Mart for Intensive Care IV database, we identified consecutive ICU patients admitted between 2008 and 2022 who fulfilled Sepsis-3 criteria and had an SIC score ≥4. BAR was calculated from the 1st available laboratory values. Multivariable Cox regression and restricted cubic splines were used to estimate mortality associations after adjustment for demographics, comorbidities, interventions, severity scores (Sequential Organ Failure Assessment, Simplified Acute Physiology Score II), and laboratory parameters. The primary end-point was 30-day mortality; 90- and 365-day mortality were secondary. Among 4244 patients, higher admission BAR independently predicted increased mortality at 30, 90, and 365 days. Each 10-unit rise in BAR conferred a 30% higher hazard of death within 30 days (unadjusted hazard ratio (HR) 1.30, 95% confidence interval (CI) 1.25–1.35; *P* < .001). After full adjustment, the association persisted (HR 1.18, 95% CI 1.12–1.24; *P* < .001). Similar patterns were seen for longer-term outcomes. A clear dose–response relationship was observed across BAR tertiles; patients in the highest tertile (BAR ≥ 10 mg/g) had markedly higher mortality risk (adjusted HR 1.69, 95% CI 1.43–2.00; *P* < .001) than those in the lowest. Nonlinear analysis revealed a curvilinear BAR–mortality relationship, and subgroup analyses showed consistent findings, with significant effect modification by age. In critically ill patients with SIC, an elevated admission BAR independently predicts short- and long-term mortality and may aid risk stratification and early intervention planning.

## 1. Introduction

Sepsis remains a major global health burden, with an estimated 48.9 million cases and 11 million deaths each year.^[1]^

Defined by the Sepsis-3 criteria, it reflects a dysregulated host response to infection that precipitates organ dysfunction^[2]^ and is characterized by systemic inflammation and progressive multi-organ failure. A frequent and serious complication is sepsis-induced coagulopathy (SIC), a spectrum of clotting disturbances – ranging from subtle laboratory changes to overt disseminated intravascular coagulation – that independently worsens outcomes.^[3]^ Roughly half of all patients with sepsis develop some degree of coagulopathy, further increasing their risk of multi-organ failure.^[4]^ The International Society on Thrombosis and Haemostasis classifies SIC as an early stage of disseminated intravascular coagulation, underscoring its potential for rapid progression without prompt intervention.^[5]^

Despite significant advances in intensive care medicine, mortality rates associated with sepsis remain persistently high,^[6–8]^ This highlights the urgent need for more effective tools for early risk stratification. Existing prognostic scoring systems often fail to fully capture the dynamic and multifaceted pathophysiology of SIC, which involves complex interactions among inflammatory activation, endothelial injury, and organ dysfunction – particularly renal impairment.^[3,4]^

Blood urea nitrogen-to-albumin ratio (BAR) has recently emerged as a promising and economically feasible biomarker that may help address this clinical gap. Importantly, the physiological correlates of BAR align closely with core mechanisms of SIC. Blood urea nitrogen (BUN) levels serve as a sensitive indicator of renal perfusion and function, both of which are frequently compromised in SIC and directly influence the clearance of uremic toxins and inflammatory cytokines.^[9–14]^ At the same time, serum albumin reflects not only nutritional status but also the degree of systemic inflammation and endothelial integrity – each being critically disrupted in SIC. As a composite metric, BAR thus integrates 2 fundamental pathological pathways – renal dysfunction and inflammatory/nutritional decline – that are central to the progression of SIC.

Although BAR has demonstrated prognostic value across a range of conditions – including COVID-19, heart failure, and acute pancreatitis,^[15–22]^ its specific relevance to SIC remains underexplored. Using the Medical Information Mart for Intensive Care IV (MIMIC-IV) database, we conducted a retrospective cohort study to examine whether BAR measured at intensive care unit (ICU) admission predicts mortality at 30, 90, and 365 days in patients with SIC. We hypothesized that higher BAR would track with increased mortality, offering a readily available metric for early risk stratification and targeted management in this high-risk group.

## 2. Methods

### 2.1. Database

Data were drawn from the MIMIC-IV database (https://physionet.org/content/mimiciv/3.1/).^[22]^ This open-access resource contains de-identified records for >40,000 critical-care admissions at Beth Israel Deaconess Medical Center, Boston, Massachusetts, and spans demographics, vital signs, laboratory values, and medication data.^[14,23,24]^

The study followed the Declaration of Helsinki. Institutional review boards at the Massachusetts Institute of Technology and Beth Israel Deaconess Medical Center approved use of MIMIC-IV. Mei Li completed the required online training and examination (Certificate ID: 69038302) to obtain access. MIMIC-IV database used in the present study was approved by the Institutional Review Boards (IRB) of Institutional Review Boards of Beth Israel Deaconess Medical Center (2001-P-001699/14) and the Massachusetts Institute of Technology (No. 0403000206) both approved the use of the database for research. The individual information of the patients included in this database was anonymous, and ethical review and informed consent were waived. We have also complied with all relevant ethical regulations regarding the use of the data for our study. Reporting conforms to the Strengthening the Reporting of Observational Studies in Epidemiology guidelines for observational studies.^[25]^

We performed a retrospective cohort analysis of MIMIC-IV v3.1, enrolling all consecutive ICU patients with SIC. The primary end-point was 30-day mortality; 90- and 365-day mortality served as secondary endpoints.

### 2.2. Study population

We screened the MIMIC-IV cohort (2008–2022) for adults (≥18 years) admitted to the ICU with SIC. Sepsis was identified using Sepsis-3 criteria – documented or suspected infection plus an acute rise in Sequential Organ Failure Assessment (SOFA) score of ≥2 points,^[1]^ and SIC was defined as an SIC score ≥4. Inclusion criteria SIC: present at ICU admission; age ≥18 years.

Exclusion criteriaI: ICU stay < 24 hours; <1 ICU admission during the hospitalization; missing admission BUN or albumin.

**Figure 1.**
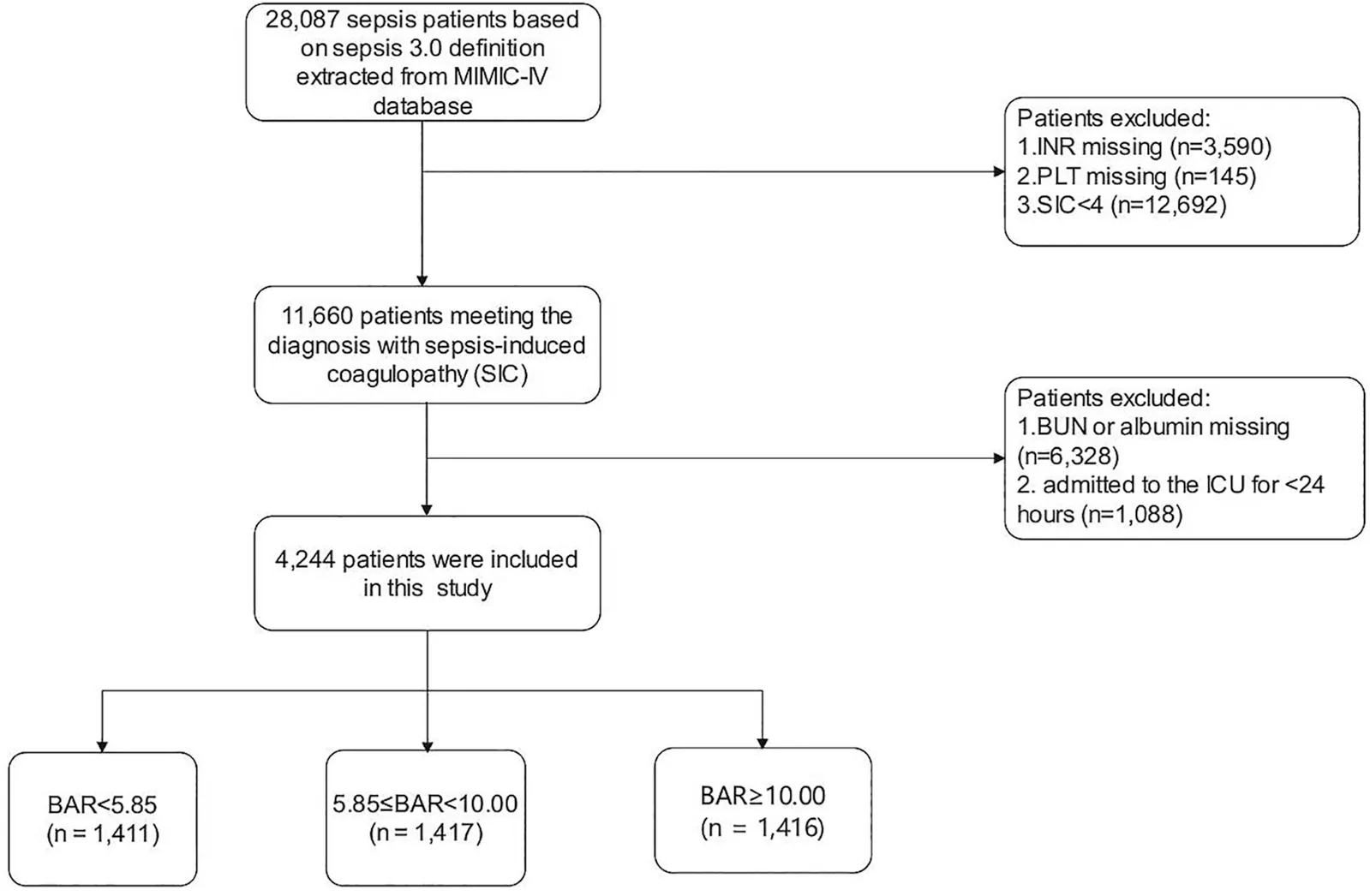
Flowchart for participants from the MIMIC-IV (v 2.2). BAR = blood urea nitrogen-to-albumin ratio, ICU = intensive care unit, INR = international normalized ratio, MIMIC-IV = Medical Information Mart for Intensive Care IV, PLT = platelets, SIC = sepsis-induced coagulopathy

The final sample comprised patients with all laboratory values required for BAR calculation available within 24 hours of ICU entry (Fig. 1).

### 2.3. Exposure

The BAR served as the primary exposure variable, analyzed both continuously and categorically. Using admission values from MIMIC-IV, BAR was stratified into tertiles: T1: <5.85 mg/g, T2: 5.85 to 9.99 mg/g, T3: ≥10.00 mg/g. Baseline BAR was defined by the initial measurement obtained within 24 hours of ICU admission.

### 2.4. Covariates

We retrieved patient-level data from MIMIC-IV with Structured Query Language, stored them in a local PostgreSQL server, and abstracted the following: demographics: age, sex, and race. Comorbidities: hypertension, diabetes mellitus, atrial fibrillation, congestive heart failure, chronic pulmonary disease, cerebrovascular disease, peripheral vascular disease, peptic ulcer disease, mild liver disease, chronic kidney disease, and malignancy. Interventions: ventilator use within 1st 24 hours, vasopressor use within 1st 24 hours, vasopressor-free days within 28 days, ventilator-free days within 28 days. Severity scores: Simplified Acute Physiology Score II (SAPS II) and SOFA scores. Admission laboratory parameters: blood urea nitrogen, serum albumin, glucose, white blood cell count, hemoglobin, platelet count. Variables with ≤20% missing data underwent multiple imputation (sample missing data overview; Table S1, Supplemental Digital Content, https://links.lww.com/MD/R646). The BAR was calculated as the quotient of initial BUN (mg/dL) and serum albumin (g/dL) based on laboratory results at ICU admission.

### 2.5. Statistical analysis

Patients were stratified into tertiles based on BAR distribution. Descriptive statistics summarized baseline characteristics. Variable normality was assessed using histograms, *Q*–*Q* plots, and Kolmogorov–Smirnov tests. Continuous variables were expressed as: mean ± standard deviation for normally distributed data.

Median with interquartile range for skewed distributions, categorical variables were reported as frequencies (%). Inter-group comparisons employed: chi-square or Fisher exact tests for categorical variables 1-way ANOVA for parametric continuous data Kruskal–Wallis test for nonparametric continuous data.

Statistical modelling: Cox proportional hazards regression models evaluated associations between BAR (continuous) and mortality outcomes. The proportional hazards assumption was verified using the scaled Schoenfeld residuals test. The results indicated no significant violation of the assumption for the global model or for the primary exposure variable BAR (Figure S1, Supplemental Digital Content, https://links.lww.com/MD/R647). Using admission values from MIMIC-IV, BAR was stratified into tertiles based on the distribution of the study cohort to ensure comparable sample sizes: T1 ≤ 5.85 mg/g, T2 5.85 to 10.00 mg/g, T3 ≥ 10.00 mg/g. Hazard ratios (HRs) with 95% confidence intervals (CIs) were computed using extended Cox models with the lowest BAR tertile as reference. Three sequential models were constructed: Model 1: unadjusted, Model 2: adjusted for demographics (age, sex, and race) and comorbidities (hypertension, diabetes, congestive heart failure, atrial fibrillation, chronic kidney disease, and chronic pulmonary disease), Model 3: additionally adjusted for physiological parameters (mean blood pressure, creatinine, potassium glucose, hemoglobin, calcium, and platelets), severity scores (SOFA and SAPS II), and interventions (24-hour ventilator/vasopressor use). Confounder selection incorporated clinical relevance, literature evidence, univariate significance (*P* < .10), and >10% effect estimate change (Table S4, Supplemental Digital Content, https://links.lww.com/MD/R646). Multicollinearity was assessed via variance inflation factor >5 indicating significance (Table S2, Supplemental Digital Content, https://links.lww.com/MD/R646).

Additionally, we applied smooth curve fitting to evaluate potential nonlinear associations between mortality outcomes and BAR. Likelihood ratio tests compared the 2-piecewise linear model against standard linear regression. Restricted cubic splines characterized exposure–response relationships and assessed linearity. As the analysis utilized the complete available dataset, no prestudy power calculation was conducted.

Survival curves generated by Kaplan–Meier analysis were compared using log-rank tests. Stratified and interaction analyses assessed the consistency of BAR–mortality associations across predefined subgroups.

Demographics: age (<65 vs ≥ 65 years), sex, and race; comorbidities: hypertension, diabetes, atrial fibrillation, congestive heart failure, chronic pulmonary disease, and chronic kidney disease; sensitivity analysis employed complete-case methodology after excluding variables with >20% missing data. Sensitivity assessments were systematically conducted to evaluate the reliability and reproducibility of the results Table S3, Supplemental Digital Content, https://links.lww.com/MD/R646). All analyses were performed using R Statistical Software (Version 4.2.2, http://www.R-project.org, The R Foundation) and Free Statistics analysis platform (Version 1.9, Beijing, China, http://www.clinicalscientists.cn/freestatistics). Statistical significance was defined as 2-tailed *P* < .05.^[26]^

## 3. Results

### 3.1. Baseline characteristics

Among 4244 ICU patients with sepsis-induced coagulopathy, BAR was divided into tertiles: T1 ≤ 5.85 mg/g, T2 5.85 to 10.00 mg/g, T3 ≥ 10.00 mg/g. Mean age was 63.8 years and 60.2% were male. Thirty-day mortality reached 32.9% (1395 deaths). Nonsurvivors entered with higher BAR and heavier comorbidity loads – most often congestive heart failure, hypertension, atrial fibrillation, diabetes, peptic ulcer disease, mild liver disease, and chronic kidney disease (Table 1).

**Table 1:** Baseline characteristics and clnical outcomes of participants classified by BAR index tertiles.

Variables	Total (n = 4244)	T1	T2	T3	*P* value
		(n = 1411)	(n = 1417)	(n = 1416)	
Demographic					
Age (yr)	63.8 ± 16.2	59.0 ± 17.1	65.6 ± 15.6	66.8 ± 14.9	**<.001**
Sex, male, n (%)	2556 (60.2)	805 (57.1)	841 (59.4)	910 (64.3)	**<.001**
Race, White, n (%)	2668 (62.9)	878 (62.2)	894 (63.1)	896 (63.3)	
Weight (kg)	84.1 ± 24.9	81.4 ± 21.3	84.3 ± 25.4	86.6 ± 27.3	**<.001**
Vital signs					
Heart rate (bpm)	90.8 ± 17.7	91.3 ± 17.2	91.0 ± 17.7	90.1 ± 18.3	.18
MBP (mm Hg)	75.8 ± 10.1	78.6 ± 10.3	75.6 ± 9.7	73.3 ± 9.6	**<.001**
Respiratory rate (bpm)	20.5 ± 4.5	20.2 ± 4.4	20.6 ± 4.6	20.7 ± 4.6	**.015**
Comobidity diseases					
Congestive heart failure, n (%)	1266 (29.8)	291 (20.6)	437 (30.8)	538 (38)	**<.001**
Cerebrovascular disease, n (%)	445 (10.5)	152 (10.8)	144 (10.2)	149 (10.5)	.868
Chronic pulmonary disease, n (%)	958 (22.6)	290 (20.6)	351 (24.8)	317 (22.4)	**.027**
Mild liver disease, n (%)	1697 (40.0)	560 (39.7)	536 (37.8)	601 (42.4)	**.041**
Renal disease, n (%)	1023 (24.1)	113 (8)	346 (24.4)	564 (39.8)	**<.001**
Peptic ulcer disease, n (%)	218 (5.1)	48 (3.4)	80 (5.6)	90 (6.4)	**.001**
Peripheral vascular disease, n (%)	488 (11.5)	129 (9.1)	182 (12.8)	177 (12.5)	**.003**
Malignant cancer, n (%)	808 (19.0)	275 (19.5)	262 (18.5)	271 (19.1)	.79
Hypertension, n (%)	2363 (55.7)	652 (46.2)	836 (59)	875 (61.8)	**<.001**
Atrial fibrillation, n (%)	1308 (30.8)	313 (22.2)	447 (31.5)	548 (38.7)	**<.001**
Diabetes, n (%)	1361 (32.1)	351 (24.9)	470 (33.2)	540 (38.1)	**<.001**
Laboratory results					
White blood cells (10^3^/μL)	14.1 ± 13.9	12.9 ± 13.9	13.9 ± 11.7	15.5 ± 15.8	**<.001**
Hemoglobin (g/dL)	9.1 ± 2.2	9.4 ± 2.3	9.1 ± 2.2	8.8 ± 2.1	**<.001**
Platelet count (10^3^/μL)	151.4 ± 114.2	152.9 ± 114.9	150.8 ± 114.8	150.6 ± 113.0	.837
Aniongap (mEq/L)	18.5 ± 6.4	16.7 ± 5.7	18.3 ± 6.2	20.6 ± 6.7	**<.001**
Calcium (mEq/L)	7.7 ± 1.0	7.8 ± 0.9	7.8 ± 1.0	7.7 ± 1.1	**.039**
Sodium (mEq/L)	135.6 ± 6.2	135.9 ± 5.4	135.8 ± 5.9	135.2 ± 7.3	**.005**
Potassium (mEq/L)	4.2 ± 0.9	3.9 ± 0.7	4.2 ± 0.8	4.6 ± 0.9	**<.001**
Creatinine (mg/dL)	2.1 ± 1.9	1.1 ± 0.7	1.9 ± 1.2	3.4 ± 2.4	**<.001**
Glucose (mg/dL)	130.0 (104.0, 178.0)	129.0 (105.0, 172.0)	132.0 (104.0, 182.0)	130.0 (103.0, 180.0)	.334
INR	19.8 (16.7, 26.8)	18.6 (16.2, 24.0)	20.2 (16.9, 27.1)	20.9 (17.2, 29.2)	**<.001**
PT (s)	2.1 ± 1.2	1.9 ± 1.0	2.1 ± 1.3	2.2 ± 1.4	**<.001**
Score system, points					
SAPS II	45.8 ± 15.9	37.4 ± 13.6	46.4 ± 14.5	53.7 ± 15.3	**<.001**
SOFA	8.3 ± 3.9	6.9 ± 3.4	8.2 ± 3.8	9.7 ± 3.9	**<.001**
Interventions					
Vasopressor-free days within 28 d (d)	17.2 ± 12.7	20.6 ± 11.1	17.5 ± 12.6	13.6 ± 13.2	**<.001**
Ventilator-free days within 28 d (d)	17.7 ± 13.0	21.4 ± 11.1	17.8 ± 13.3	13.8 ± 13.5	**<.001**
Vasopressor use within 1st 24 h (h)	1867 (44.0)	631 (44.7)	642 (45.3)	594 (41.9)	.158
Ventilator use within 1st 24 h (h)	1939 (45.7)	563 (39.9)	642 (45.3)	734 (51.8)	**<.001**
Outcomes					
30-d mortality, n (%)	1395 (32.9)	285 (20.2)	456 (32.2)	654 (46.2)	**<.001**
90-day mortality, n (%)	1708 (40.2)	372 (26.4)	554 (39.1)	782 (55.2)	**<.001**
365-day mortality, n (%)	2075 (48.9)	488 (34.6)	683 (48.2)	904 (63.8)	**<.001**

Data are presented as the mean, standard deviation, interquartile range or number (%), as appropriate.

BAR = blood urea nitrogen-to-albumin ratio, INR = international normalized ratio, MBP = mean blood pressure, PT = prothrombin time, SAPS II = Simplified Acute Physiology Score II, SIC = sepsis-induced coagulopathy, SOFA = Sequential Organ Failure Assessment.

*P* values <.05 is shown in bold.

### 3.2. Clinical outcomes

Multivariable Cox modeling showed that a higher admission BAR remained independently associated with 30-day mortality (Table 2). In the crude analysis (Model 1) each 10 mg/g rise in BAR carried a 30% increase in death risk (HR 1.30, 95% CI 1.25–1.35; *P* < .001). The signal stayed consistent after stepwise adjustment:

**Table 2:** Univariate and multivariate Cox regression analyses of the association between BAR and 30-, 90-, and 365-day mortality in patients with SIC.

Variable	Crude model		Model I		Model II		Model III	
	HR (95% CI)	*P* value	HR (95% CI)	*P* value	HR (95% CI)	*P* value	HR (95% CI)	*P* value
30-d-mordality								
BAR (continues)	1.3 (1.25–1.35)	<.001	1.28 (1.24–1.33)	<.001	1.31 (1.26–1.36)	<.001	1.18 (1.12–1.24)	<.001
Tertile								
T1 (BAR < 5.85)	1 (Ref)		1 (Ref)		1 (Ref)		1 (Ref)	
T2 (5.85 ≤ BAR < 10.00)	1.7 (1.47–1.97)	<.001	1.57 (1.35–1.82)	<.001	1.65 (1.42–1.92)	<.001	1.20 (1.03–1.40)	.023
T3 (BAR ≥ 10.00)	2.75 (2.39–3.16)	<.001	2.52 (2.19–2.91)	<.001	2.84 (2.46–3.29)	<.001	1.69 (1.43–2.00)	<.001
*P* for trend		<.001		<.001		<.001		<.001
90-d-mordality								
BAR (continues)	1.3 (1.26–1.35)	<.001	1.29 (1.24–1.33)	<.001	1.31 (1.27–1.36)	<.001	1.20 (1.15–1.26)	<.001
Tertile								
T1 (BAR < 5.85)	1 (Ref)		1 (Ref)		1 (Ref)		1 (Ref)	
T2 (5.85 ≤ BAR < 10.00)	1.62 (1.42–1.85)	<.001	1.48 (1.30–1.69)	<.001	1.56 (1.37–1.79)	<.001	1.18 (1.03–1.35)	.017
T3 (BAR ≥ 10.00)	2.64 (2.33–2.99)	<.001	2.41 (2.13–2.74)	<.001	2.7 (2.37–3.07)	<.001	1.71 (1.47–1.98)	<.001
*P* for trend		<.001		<.001		<.001		<.001
365-d-mordality								
BAR (continues)	1.29 (1.25–1.33)	<.001	1.27 (1.23–1.32)	<.001	1.30 (1.25–1.34)	<.001	1.20 (1.14–1.25)	<.001
Tertile								
T1 (BAR < 5.85)	1 (Ref)		1 (Ref)		1 (Ref)		1 (Ref)	
T2 (5.85 ≤ BAR < 10.00)	1.56 (1.39–1.75)	<.001	1.41 (1.25–1.59)	<.001	1.48 (1.31–1.66)	<.001	1.15 (1.02–1.30)	.025
T3 (BAR ≥ 10.00)	2.46 (2.21–2.75)	<.001	2.23 (2.00–2.5)	<.001	2.44 (2.18–2.75)	<.001	1.61 (1.41–1.84)	<.001
*P* for trend		<.001		<.001		<.001		<.001

Crude model: unadjusted.

Model 1: Adjusted age, sex, race.

Model 2: Adjusted Model 1 plus weight, hypertension, diabetes, congestive, heart failure, atrial fibrillation, renal disease, chronic pulmonary.

Model 3: Adjusted Model 2 plus MBP, creatinine, potassium, glucose, hemoglobin, calcium, platelet, SOFA, SAPS II, Vasopressor use within 1st 24 hours.

Ventilator use within 1st 24 hours.

BAR = blood urea nitrogen-to-albumin ratio, CI = confidence interval, HR = hazard ratio, MBP = mean blood pressure, SAPS II = Simplified Acute Physiology Score II, SIC = sepsis-induced coagulopathy, SOFA = Sequential Organ Failure Assessment.

Model 1: demographics only (age, sex, and race).

Model 3: plus comorbidities (hypertension, diabetes, congestive heart failure, atrial fibrillation, chronic pulmonary disease, and chronic kidney disease), early interventions (ventilator or vasopressor within 24 hours), admission laboratories (creatinine, potassium, glucose, hemoglobin, calcium, and platelets), severity scores (SOFA and SAPS II) and weight (HR 1.18, 95% CI 1.12–1.24; *P* < .001).

Using BAR tertiles and T1 (≤5.85 mg/g) as reference:

T2 (5.85–10.00 mg/g): adjusted HR 1.20 (95% CI 1.03–1.40; *P* = .021)

T3 (≥10.00 mg/g): adjusted HR 1.69 (95% CI 1.43–2.00; *P* < .001)

A clear dose-response gradient spanned the tertiles.

### 3.3. Long-term mortality associations

Elevated admission BAR consistently predicted increased mortality risk across timeframes. For 90-day mortality: Model 1 (unadjusted): HR 1.30 per 10 mg/g BAR increase (95% CI 1.26–1.35; *P* < .001), Model 3 (fully adjusted): HR 1.20 (95% CI 1.15–1.26; *P* < .001), compared to T1 (≤5.85 mg/g): T2: adjusted HR 1.18 (95% CI 1.03–1.35; *P* = .018) T3: adjusted HR 1.71 (95% CI 1.47–1.98; *P* < .001). For 365-day mortality: Model 1: HR 1.29 (95% CI 1.25–1.33; *P* < .001), Model 3: HR 1.20 (95% CI 1.14–1.25; *P* < .001) versus T1: T2: adjusted HR 1.15 (95% CI 1.02–1.30; *P* = .023), T3: adjusted HR 1.61 (95% CI 1.41–1.84; *P* < .001) (Table 2).

**Figure 2.**
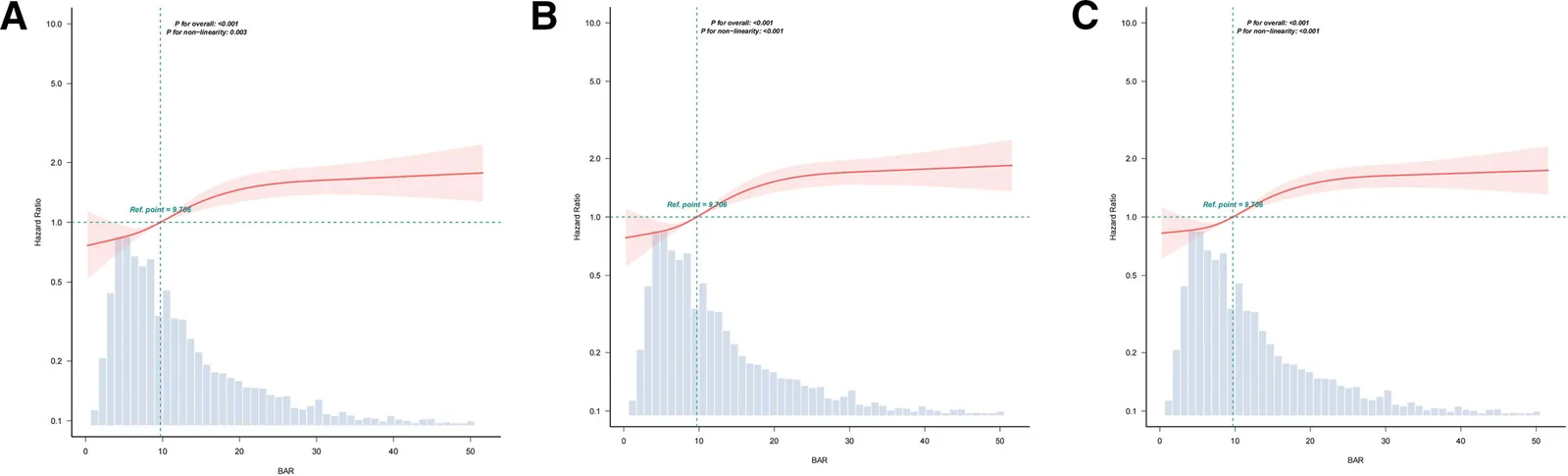
Restricted cubic spline (RCS) curves illustrating the multivariable-adjusted hazard ratios for all-cause mortality at (A) 30 days, (B) 90 days, and (C) 365 days according to BAR levels. The nonlinear relationship between BAR levels and the risk mortality was analyzed, with adjustment for age, sex, race, weight, hypertension, diabetes, congestive, and heart failure, atrial fibrillation, renal disease, chronic pulmonary, plus MBP, creatinine, potassium, glucose, hemoglobin, calcium, platelet, SOFA, SAPS II, and vasopressor use within 1st 24 hours, ventilator use within 1st 24 hours. BAR = blood urea nitrogen-to-albumin ratio. MBP = mean blood pressure, SAPS II = Simplified Acute Physiology Score II, SOFA = Sequential Organ Failure Assessment.

Curve fitting analysis revealed a nonlinear relationship between BAR levels and 30, 90, 365 day mortality risk (*P* nonlinearity = .009; Fig. 2). Based on graphical interpretation and clinical relevance, we identified an inflection point at 27.34 mg/g. Below this threshold, each 1 mg/g increase in BAR was associated with a 3.8% higher mortality risk (HR 1.038, 95% CI 1.027–1.049; *P* < .001). Above 27.34 mg/g, no significant association was observed (HR 0.994, 95% CI 0.959–1.031; *P* = .753) (Table 3).

**Table 3:** Threshold effect analysis of the relationship of BAR with 30-day mortality.

Threshold of BAR	HR	95% CI	*P* value
<27.34	1.0380	1.0270–1.0490	<.001
≥27.34	0.9942	0.9589–1.0308	.7533
Nonlinear test			.009

BAR = blood urea nitrogen-to-albumin ratio, CI = confidence interval, HR = hazard ratio.

### 3.4. Kaplan–Meier survival curve analysis

**Figure 3.**
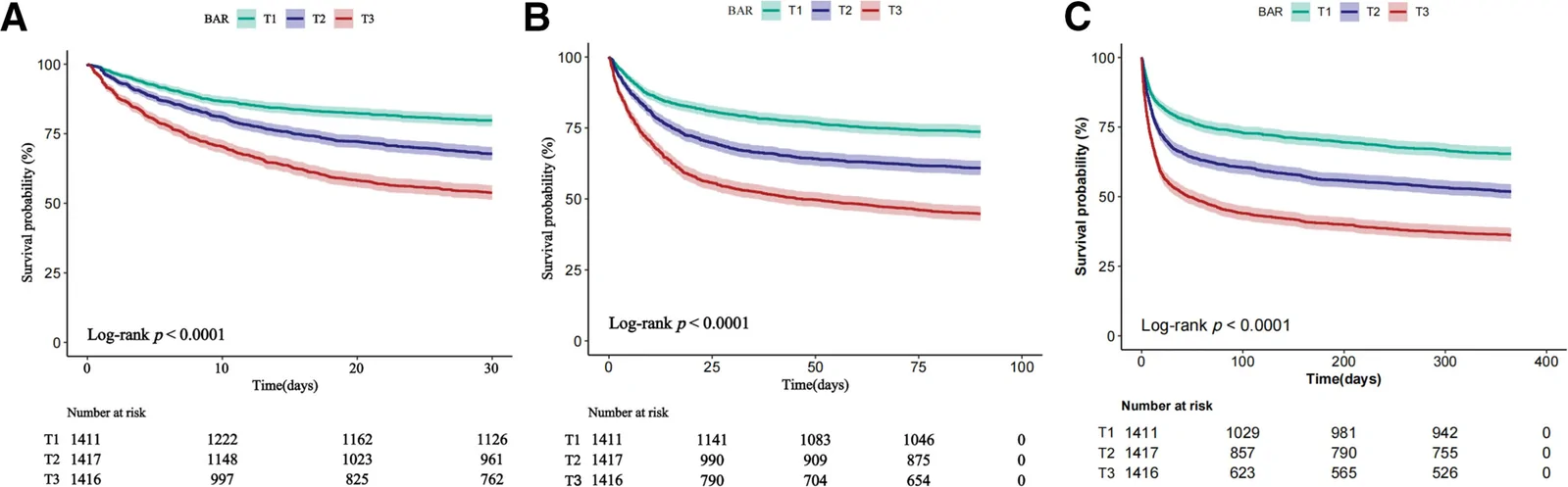
Kaplan–Meier survival curves for all-cause mortality stratified by BAR. (A) 30-day mortality, (B) 90 day mortality, and (C) 365 day mortality. BAR = blood urea nitrogen-to-albumin ratio.

Figure 3 depicts Kaplan–Meier curves stratified by BAR tertiles. The T3 group (BAR ≥ 10.00 mg/g) demonstrated significantly lower 30-day survival probability compared to T1 (BAR ≤ 5.85 mg/g) (log-rank *P* < .0001).

### 3.5. Subgroup analysis

**Figure 4.**
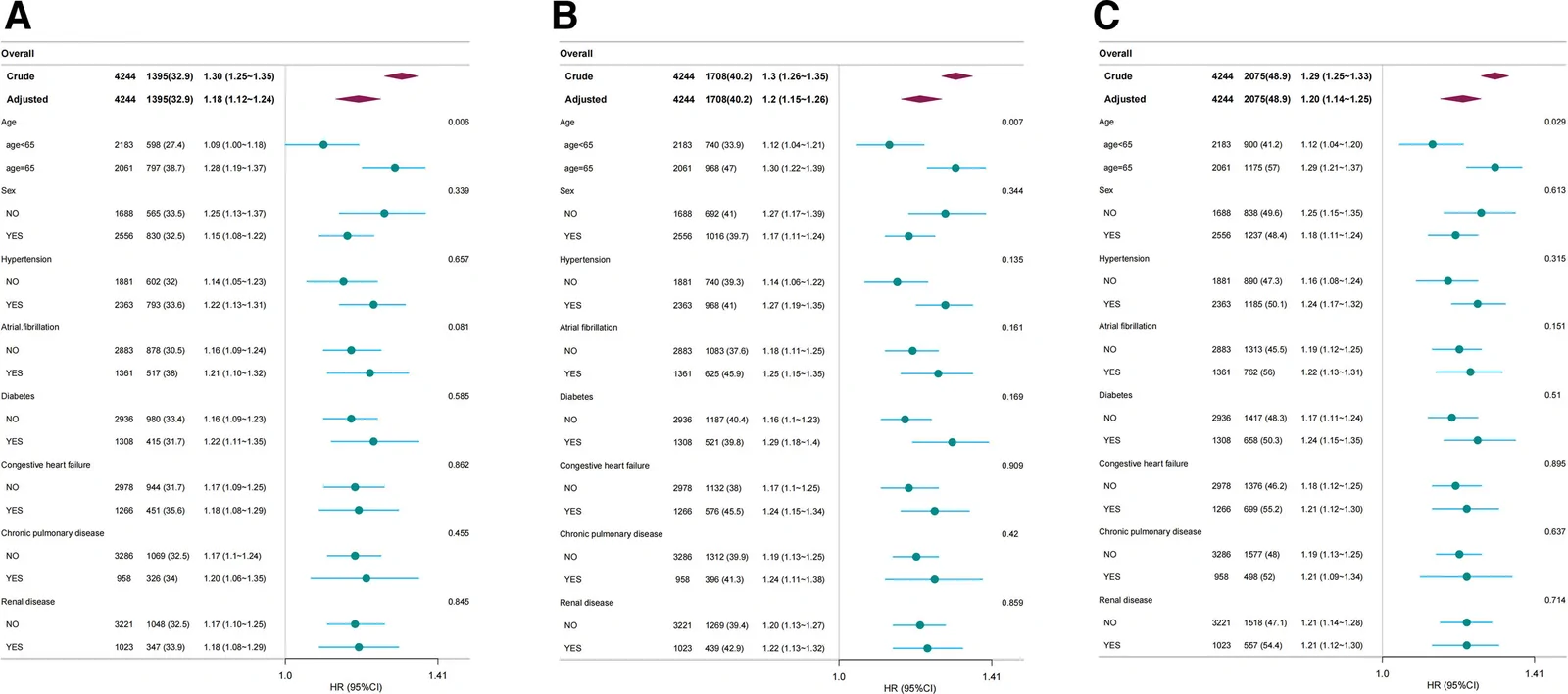
Subgroup analyses for the association of BAR levels with all-cause mortality at (A) 30 days, (B) 90 days, and (C) 365 days. Exploratory post hoc subgroup analyses were performed using multivariable-adjusted Cox regression models to estimate hazard ratios (HRs) and 95% confidence intervals (CIs) across key clinical subgroups, including age, sex, race, hypertension, atrial fibrillation, diabetes, congestive heart failure, chronic pulmonary disease, renal disease. Adjustment for age, sex, race, weight, hypertension, diabetes, congestive, heart failure, atrial fibrillation, renal disease, chronic pulmonary plus MBP, creatinine, potassium, glucose, hemoglobin, calcium, platelet, SOFA, SAPS II, vasopressor use within 1st 24 hours, ventilator use within 1st 24 hours. *P* values for interaction were calculated to assess potential effect modification. MBP = mean blood pressure, SAPS II = Simplified Acute Physiology Score II, SOFA = Sequential Organ Failure Assessment.

Consistent associations between elevated BAR and increased mortality were observed across all predefined subgroups (age, sex, hypertension, diabetes, atrial fibrillation, congestive heart failure, chronic pulmonary disease, and chronic kidney disease; Fig. 4). Significant effect modification occurred by age stratification (<65 vs ≥ 65 years; *P* interaction < .05).

### 3.6. Sensitivity analysis

To verify the stability of the main results, we conducted sensitivity analysis. Sensitivity analysis was conducted using multivariable cox regression models applied to datasets before and after multiple imputation of missing values. The link between BAR and mortality at 30-, 90-, and 365-day remained robust and aligned with the primary findings (Table S3, Supplemental Digital Content, https://links.lww.com/MD/R646).

## 4. Discussion

We found that a higher BAR at admission tracked tightly with rising 30-, 90-, and 365-day mortality in ICU patients with SIC. Cox models showed that every 10 mg/g increment in BAR translated into a 30% increase in 30-day death (unadjusted HR 1.30, 95% CI 1.25–1.35; *P* < .001), and the signal barely weakened after stepwise adjustment: Model 1 HR 1.28 (1.24–1.33), Model 3 HR 1.18 (1.12–1.24). When split into tertiles, patients in T3 (BAR ≥ 10.00 mg/g) carried an adjusted 30-day hazard of 1.69 (1.43–2.00) relative to T1 (≤5.85 mg/g), with T2 showing an intermediate 1.20 (1.03–1.40); the same graded pattern held for longer-term outcomes. Restricted cubic spline analysis revealed a curvilinear dose–response (Fig. 2), and Kaplan–Meier curves confirmed markedly lower 30-day survival in T3 (log-rank *P* < .0001; Fig. 3). BAR remained informative across clinically relevant subgroups (Fig. 4), although the prognostic impact was amplified in patients aged ≥65 years (*P* interaction = .032). These data establish BAR as an independent mortality indicator in SIC – an observation not previously reported.

BAR prognostic punch in SIC stems from the combined biology of its 2 constituents. BUN mirrors renal clearance: when glomerular filtration falters, nitrogenous waste accumulates and the number rises. In SIC, acute kidney injury is common and each upward tick of BUN usually signals deeper involvement of the kidneys in the septic cascade – driven by hypoperfusion, inflammation and endothelial injury – that in turn amplifies coagulopathy and multi-organ failure. Albumin occupies the opposite pole; it is a read-out of hepatic synthetic capacity, nutritional reserve and the intensity of systemic inflammation. Hypoalbuminaemia is therefore routine in sepsis, where capillary leak, catabolism and cytokine storm deplete the protein and simultaneously blunt immune responses and endothelial integrity. The lower the albumin, the richer the inflammatory milieu and the more vigorous the coagulation dysfunction.^[27–31]^ By coupling these 2 pathways, BAR integrates renal injury, nutritional deficit and inflammatory stress into a single bedside number. In practice it captures the intersection of organ dysfunction, coagulopathy and metabolic derangement that decides prognosis in SIC – offering an early alert for patients slipping toward adverse outcomes.^[32,33]^Our large-scale cohort of 4244 patients with SIC confirms and extends the prognostic utility of the BAR. We found that elevated BAR at admission independently predicted higher short- and long-term mortality, even after extensive adjustment for illness severity scores. Importantly, our analysis identified a nonlinear relationship between BAR and mortality, with a critical inflection point at 27.34 mg/g, and revealed patient subgroups in which BAR carries particular prognostic weight. Together, these findings reinforce the view that BAR reflects a composite of pathophysiological disturbances relevant to SIC.

The dose-dependent association between BAR and mortality at 30, 90, and 365 days highlights its value as an indicator of systemic physiological decompensation. That this association persisted after adjustment for SOFA and SAPS II scores suggests BAR provides prognostic information beyond conventional severity measures. This is consistent with the understanding that BAR integrates 2 key biological pathways: albumin as a marker of chronic reserve and BUN levels as a signal of acute insults such as inflammation, hypoperfusion, inflammation, and endothelial injury.^[19,20,34–45]^ Our results lend strong support to the previously proposed “chronic-baseline–acute-on-top” axis.^[35,38,42]^ The higher comorbidity burden among nonsurvivors, along with significant effect modification by age (*P* interaction < .05), indicates that BAR prognostic association is heightened in patients with limited physiological reserve, reinforcing that a given BAR increase conveys greater risk in older or chronically ill patients.^[35]^

A key advance of our study is the clarification of a nonlinear BAR–mortality relationship, with an inflection point at 27.34 mg/g. This moves beyond earlier suggestions of a J-shaped association^[20]^ by providing a precise threshold. Below this value, risk increased steeply with rising BAR (HR 1.038 per 1 mg/g), consistent with progressive dysregulation along an “acute uncontrolled-inflammation” pathway.^[20,34,35]^ Above it, however, risk plateaued – offering empirical support for the concept of “hepatic immune paralysis” and implying that patients beyond this threshold may have entered a state of irreversible physiological failure. This distinction has clear implications for risk stratification, helping to identify a very high-risk subgroup that may merit tailored management. BAR ability to predict both early and late mortality further suggests it captures not only the initial insult but also prolonged physiological decline. Although most patients in our cohort had bacterial sepsis, the strength of the BAR–mortality link in SIC – a condition involving intertwined inflammatory, coagulopathic, and endothelial dysfunction – hints that BAR may also be sensitive to microvascular injury, potentially extending its relevance to hemolytic and host-response pathways.^[4,36,37,39]^

An interesting and novel finding in our study is the significant age-by-BAR interaction, which revealed that the prognostic value of BAR was more pronounced in patients <65 years. This interaction suggests that age modifies the relationship between BAR and mortality, with younger patients showing a more robust association between elevated BAR and increased mortality risk. This phenomenon may be attributed to several factors. In younger patients, SIC may primarily be driven by renal dysfunction and nutritional depletion, both of which are captured well by BAR. Additionally, younger individuals may have fewer comorbidities, such as chronic cardiovascular or renal diseases, which could mitigate the impact of renal and inflammatory derangements in sepsis. In contrast, older patients often present with multimorbidity, including chronic kidney disease, cardiovascular disease, and immune senescence, all of which may diminish the predictive power of BAR for mortality. Therefore, while BAR remains an important predictor in older individuals, the presence of other comorbidities and age-related physiological changes might dilute its relationship with outcomes in this group.

In summary, our study strengthens the case for BAR as a key prognostic marker in SIC. We corroborate previously proposed risk thresholds (such as BAR ≥ 10.00 mg/g, corresponding to our highest-risk tertile) while refining the underlying model by establishing a critical inflection point and demonstrating important effect modification by age. These results support the incorporation of BAR into routine risk assessment in SIC. Moreover, the clear delineation of a high-risk subgroup based on BAR supports its use as an enrichment marker in future trials of adjuvant therapies, especially those targeting immune dysregulation in severe sepsis. Our study has several important clinical implications. The results underscore that BAR, a simple and cost-effective biomarker, can be used for early risk stratification in critically ill patients with SIC. Given that BAR is derived from routine laboratory measurements (BUN and albumin), it can be easily incorporated into clinical practice to aid in identifying high-risk patients who may benefit from more aggressive monitoring and intervention. For patients with elevated BAR, clinicians may consider early renal support and targeted interventions aimed at reducing systemic inflammation. For instance, timely renal replacement therapy could be considered in patients with high BAR and evidence of acute kidney injury. Moreover, early nutritional support and antiinflammatory treatments could help mitigate the effects of low serum albumin, potentially improving patient outcomes. Incorporating BAR into multimodal scoring systems (e.g., combining it with SOFA or SAPS II) could further enhance its utility, providing a comprehensive view of a patient prognosis and aiding in personalized care decisions.

This study has several limitations that should be considered. First, as a retrospective analysis using the MIMIC-IV database from a single tertiary care center, the generalizability of our findings – including the identified BAR thresholds (T3 ≥ 10.00 mg/g and the inflection point at 27.34 mg/g) – may be limited when applied to populations with different demographic characteristics or healthcare settings. Second, reliance on a single baseline BAR measurement obtained within 24 hours of ICU admission provides only a static snapshot and cannot capture dynamic changes in the ratio that might reflect disease progression or response to therapy. Third, despite multivariate adjustment for known confounders, residual confounding from unmeasured factors such as detailed treatment decisions or genetic backgrounds cannot be entirely excluded. Fourth, the nonlinear relationship between BAR and mortality, while significant, lacks mechanistic explanation; the biological significance of the inflection point remains unclear. Finally, as a composite measure, BAR does not allow disentanglement of the individual contributions of its components (BUN and albumin) to patient outcomes. Despite these limitations, our findings support the prognostic value of BAR and highlight the need for future prospective, multi-center studies to validate its thresholds, explore its dynamic trends, and elucidate the underlying mechanisms, particularly concerning the 27.34 mg/g inflection point. While acknowledging the study constraints, our findings provide a solid foundation for advancing BAR application in critical care settings. The integration of multivariable adjustments, sensitivity analyses, and demonstration of dose–response relationships supports the robustness of our conclusions, paving the way for BAR potential integration into clinical practice. Building on this work, future research should prioritize: external validation of BAR thresholds through prospective, multi-center studies; longitudinal investigations to characterize the dynamic behavior of BAR and its clinical implications; integrated mechanistic studies that combine BAR with multi-omics data (e.g., inflammatory cytokines and endothelial markers) to elucidate the drivers of the relationship, particularly the significance of the 27.34 mg/g inflection point. The ultimate objective is to evolve BAR from a prognostic marker into a tool that actively informs clinical decision-making.

## 5. Conclusion

This study shows that an elevated admission BAR is an independent predictor of higher 30-, 90-, and 365-day mortality in surgical intensive care patients. Incorporating BAR into early risk stratification could help flag those most at risk and guide tailored interventions. By coupling blood urea nitrogen – an index of renal function – with albumin, a marker of inflammatory-nutritional status, clinicians gain a dual-pathway snapshot that may sharpen critical-care decision-making. However, large-scale prospective studies are needed to validate these findings before BAR can be widely adopted in routine clinical practice. Future studies should use randomized controlled trials to establish causality and explore mechanisms linking BAR levels to SIC outcomes, including inflammatory, coagulopathic, and endothelial dysfunction.

## Acknowledgments

We acknowledge the Massachusetts Institute of Technology and Beth Israel Deaconess Medical Centre for providing access to the MIMIC-IV database. We thank Dr Jie Liu (Chinese People’s Liberation Army General Hospital, Beijing) and Dr Qilin Yang (Second Affiliated Hospital of Guangzhou Medical University) for their scholarly critique during manuscript revision.

## Author contributions

**Conceptualization:** Mei Li, Huibo Wang.

**Data curation:** Huibo Wang.

**Formal analysis:** Mei Li.

**Funding acquisition:** Hua Wang, Zhaojun Xu.

**Investigation:** Huibo Wang.

**Methodology:** Mei Li.

**Project administration:** Zhaojun Xu.

**Software:** Mei Li.

**Supervision:** Hua Wang.

**Validation:** Huibo Wang.

**Visualization:** Hua Wang.

**Writing – original draft:** Mei Li.

## Supplementary Material




