# Supplementary material for: Association of blood urea nitrogen to serum albumin ratio with mortality in ICU patients with sepsis-induced coagulopathy: A Retrospective Cohort Study from MIMIC-IV database
Source: Medicine (Baltimore). 2026 Apr 10;105(15):e48296. doi: 10.1097/MD.0000000000048296 (PMC13592927; doi:10.1097/MD.0000000000048296)
Supplement: Supplementary file 1 [file medi-105-e48296-s001.pdf]

■ Supplemental Table 1 Sample missing data overview

Variable	Valid Samples	Missing Samples	Missing Ratios
**Demographic**			
Age	4244	0	0
Sex	4244	0	0
Race	4244	0	0
Weight	4207	37	0.8718
**Vital signs**			
Respiration rate	4243	1	0.0236
MBP	4244	0	0
Heart rate	4244	0	0
**Laboratory results**			
Anion gap	4242	2	0.0471
Calcium	4208	36	0.8483
Creatinine	4244	0	0
Albumin	4240	4	0
Glucose	4242	2	0.0471
INR	4244	0	0
Platelet	4244	0	0
Potassium	4241	3	0.0707
Blood Urea nitrogen	4244	0	0
White blood cells	4239	5	0.1178
Hemoglobin	4241	3	0.0707
PT	4244	0	0
Sodium	4243	1	0.0236
**Comobidity diseases**			
Cerebrovascular	4244	0	0

Congestive heart failure	4244	0	0
Diabetes	4244	0	0
Hypertension	4244	0	0
Atrial fibrillation	4244	0	0

Supplemental Table 2 Variance inflation factors (VIFs) for covariates included in the Multivariable cox regression models.

Variable	VIF
Age	1.866
Sex	1.118
Race	1.060
Weight	1.224
Hypertension	1.232
Diabetes	1.235
Atrial fibrillation	1.319
Congestive heart failure	1.254
Renal disease	1.297
Chronic pulmonary disease	1.046
MBP	1.148
Creatinine	1.939
Potassium	1.168
Glucose	1.158
Hemoglobin	1.139
Calcium	1.088
Platelet count	1.257
SAPSII	2.835
SOFA	4.017
Vasopressor use within first 24 hours(hours)	1.207
Ventilator use within first 24 hours(hours)	1.599

Supplemental Table 3 Univariate and Multivariate cox regression analyses of the association between BAR and 30-, 90-, and 365-Day mortality in Patients with SIC after excluding patients missing the blood urea nitrogen and albumin ratio

Variable	Crude model		Model I		Model II		Model III	
	HR (95%CI)	*P-value*	HR (95%CI)	*P-value*	HR (95%CI)	*P-value*	HR (95%CI)	*P-value*
30-day-mordality								1
BAR (continues)	1.3 0(1.25~1.34)	<0.001	1.28 (1.23~1.33)	<0.001	1.31 (1.26~1.36)	<0.001	1.18 (1.12~1.24)	<0.001
Tertile								
T1(0.26-5.85)	1 (Ref)		1(Ref)		1 (Ref)		1(Ref)	
T2 (5.85-10.00)	1.72 (1.49~2.00)	< 0.001	1.59 (1.36~1.84)	< 0.001	1.67 (1.43~1.94)	< 0.001	1.21 (1.04~1.42)	0.015
T3(10.00-107.33)	2.79 (2.42~3.21)	< 0.001	2.55 (2.21~2.94)	< 0.001	2.86 (2.47~3.32)	< 0.001	1.72 (1.46~2.04)	< 0.001
P for trend		< 0.001		< 0.001		< 0.001		< 0.001
90-day-mordality								
BAR (continues)	1.3 (1.26~1.34)	< 0.001	1.28 (1.24~1.33)	< 0.001	1.31 (1.27~1.36)	< 0.001	1.20 (1.15~1.26)	< 0.001
Tertile								
T1(0.26-5.85)	1 (Ref)		1 (Ref)		1 (Ref)		1(Ref)	
T2 (5.85-10.00)	1.63 (1.43~1.87)	< 0.001	1.49 (1.3~1.71)	< 0.001	1.57 (1.37~1.80)	< 0.001	1.19 (1.04~1.37)	0.012
T3(10.00-107.33)	2.65 (2.34~3.00)	< 0.001	2.41 (2.13~2.74)	< 0.001	2.69 (2.36~3.07)	< 0.001	1.73 (1.49~2.01)	< 0.001
P for trend		< 0.001		< 0.001		< 0.001		< 0.001
365-day-mordality								
BAR (continues)	1.29 (1.25~1.33)	< 0.001	1.27 (1.23~1.31)	< 0.001	1.29 (1.25~1.33)	< 0.001	1.20 (1.15~1.25)	< 0.001
Tertile								
T1(0.26-5.85)	1 (Ref)		1 (Ref)		1 (Ref)		1(Ref)	
T2 (5.85-10.00)	1.56 (1.39~1.75)	< .001	1.41 (1.25~1.58)	< 0.001	1.47 (1.30~1.65)	< 0.001	1.16 (1.02~1.31)	0.021
T3(10.00-107.33)	2.46 (2.20~2.75)	< 0.001	2.22 (1.98~2.49)	< 0.001	2.42 (2.15~2.72)	< 0.001	1.63 (1.43~1.87)	< 0.001

P for trend	< 0.001	< 0.001	< 0.001	< 0.001

■ Supplemental Table 4 Results of Univariate cox regression analysis

Variables	Univariable COX		*P*-value
	HR	95%CI	
**Demographic**			
Age (years)	1.02	1.01,1.02	< 0.001
Sex,male, n (%)	0.97	0.88,1.08	0.626
Race, white,n (%)	1.27	1.14,1.41	< 0.001
Weight (kg)	1.00	0.99,1.00	0.941
**Vital signs**			
Heart rate (bpm)	1.00	1.00,1.01	< 0.001
MBP (mmHg)	0.97	0.97,0.98	< 0.001
Respiratory rate (bpm)	1.06	1.05,1.07	< 0.001
**Comobidity diseases**			
Congestive heart failure, n (%)	1.14	1.02,1.28	0.018
Cerebrovascular disease, n (%)	1.44	1.24,1.68	< 0.001
Chronic pulmonary disease, n (%)	1.05	0.93,1.19	0.402
Mild liver disease, n (%)	1.16	1.04,1.29	0.006
Renal disease, n (%)	1.04	0.92,1.17	0.572
Peptic ulcer disease, n (%)	0.85	0.66,1.09	0.199
Peripheral vascular disease, n (%)	1.08	0.92,1.27	0.331
Malignant cancer, n (%)	1.47	1.3,1.66	< 0.001
Hypertension, n (%)	1.04	0.94,1.16	0.417
Diabetes, n (%)	0.93	0.83,1.04	0.223
Atrial fibrillation, n (%)	1.29	1.15,1.43	< 0.001
**Laboratory results**			
White blood cells (10^3/μL)	1.01	1.00,1.01	< 0.001
Hemoglobin (g/dL)	0.97	0.94,0.99	0.005
Platelet count (10^3/μL)	1.00	0.99,1.00	0.47
Aniongap (mEq/L)	1.07	1.06,1.07	< 0.001

Calcium (mEq/L)	0.99	0.93,1.04	0.622
Sodium (mEq/L)	0.99	0.98,1.00	0.174
Potassium (mEq/L)	1.26	1.19,1.33	< 0.001
Creatinine (mg/dL)	1.08	1.06,1.11	< 0.001
Glucose (mg/dL)	1.00	0.99,1.00	0.349
Blood urea nitrogen(mg/dL)	1.00	1.00,1.01	< 0.001
Serum albumin (g/dL)	0.79	0.73,0.86	< 0.001
BAR(mg/g)	1.03	1.02,1.03	< 0.001
INR	1.11	1.08,1.14	< 0.001
PT (second)	1.01	1.01,1.01	< 0.001
**Score system,points**			
SAPSII	1.04	1.04,1.05	< 0.001
SOFA	1.15	1.13,1.16	< 0.001
**Interventions**			
Ventilator-free days within 28 days(days)	0.91	0.90,0.91	< 0.001
Vasopressor-free days within 28 days(days)	0.95	0.95,0.95	< 0.001
Ventilator use within first 24 hours(hours)	1.36	1.23,1.51	< 0.001
Vasopressor use within first 24hours(hours)	1.57	1.41,1.75	< 0.001
