# Supplementary material for: Association of blood urea nitrogen to serum albumin ratio with mortality in ICU patients with sepsis-induced coagulopathy: A Retrospective Cohort Study from MIMIC-IV database
Source: Medicine (Baltimore). 2026 Apr 10;105(15):e48296. doi: 10.1097/MD.0000000000048296 (PMC13592927; doi:10.1097/MD.0000000000048296)
Supplement: Supplementary file 2 [file medi-105-e48296-s002.pdf]

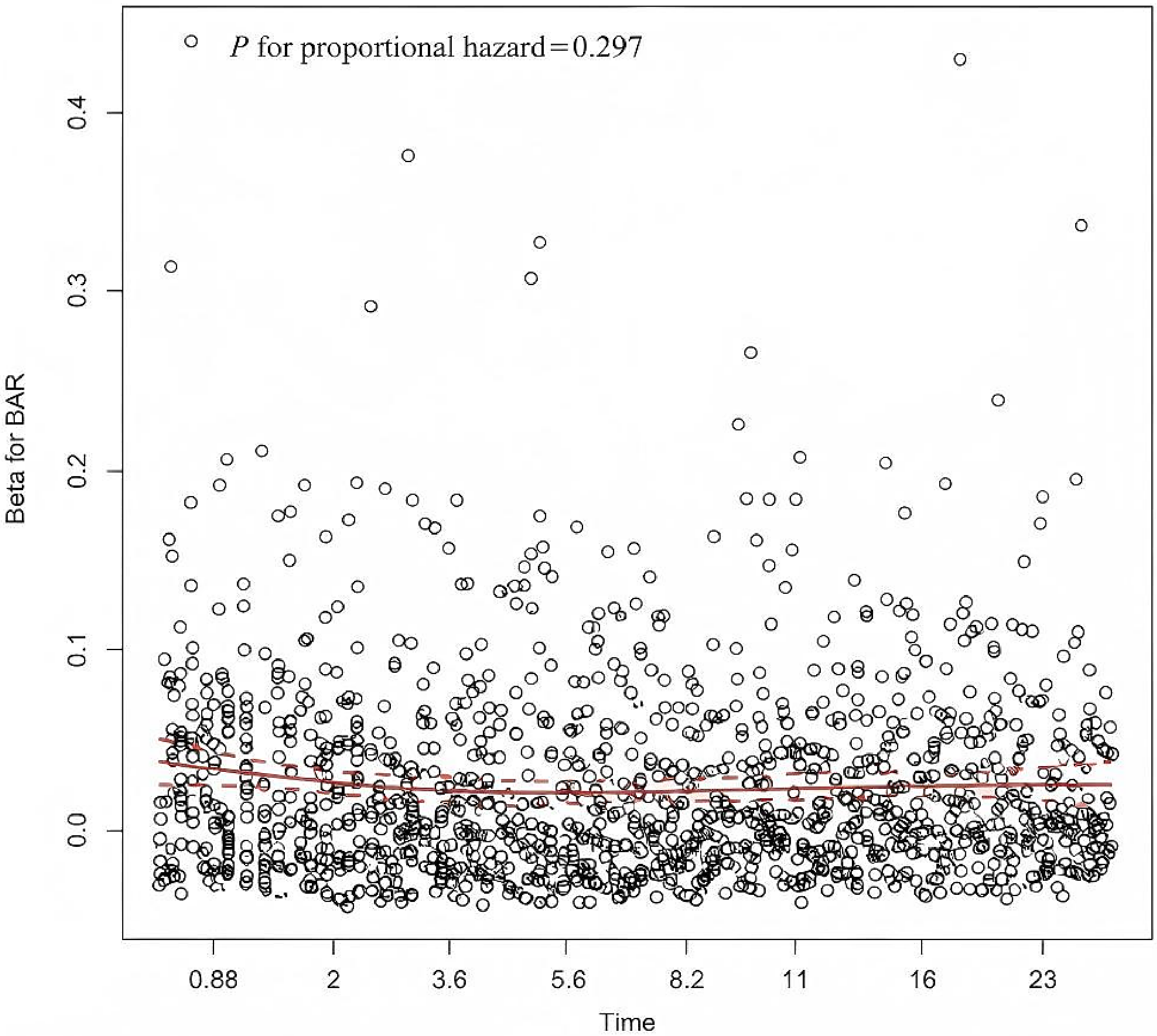

Supplemental Figure: Proportional hazards assumption test result. Scatter plot of scaled Schoenfeld residuals over time for the BAR variable. P for proportional hazard=0.297.
